# Association of perceived stress and sleep quality among medical students: the mediating role of anxiety and depression symptoms during COVID-19

**DOI:** 10.3389/fpsyt.2024.1272486

**Published:** 2024-01-18

**Authors:** Wanmin Huang, Xueke Wen, Yunjia Li, Chunliu Luo

**Affiliations:** ^1^Guangzhou Panyu Central Hospital, Guangzhou, China; ^2^School of Nursing, Jinan University, Guangzhou, China; ^3^Medical Imaging Center, The First Affiliated Hospital, Jinan University, Guangzhou, Guandong, China

**Keywords:** medical student, perceived stress, sleep quality, anxiety, depression

## Abstract

COVID-19 has intensified the influence on medical students by changing the lifestyle, online study and clinical practice which bring out series of stress, sleep disturbance and mental health problems. This cross-sectional study aim to explore the association between perceived stress and sleep quality among medical students and investigated whether anxiety and depression mediated this association during pandemic. A total of 1,021 medical students in two universities who were from Guangdong Province, China participated this study and from January to September 2020 through the online question are, with 28.80% reporting sleep disturbances. The medical students were finished various self-reported questionnaires, including the Perceived Stress Scale-10, Pittsburgh Sleep Quality Index, Generalized Anxiety Disorder-7 Scale, and Patient Health Questionnaire-9, the study found positive correlations among sleep quality, perceived stress, anxiety, and depression. The data was analyzed with The Amos 26.0 system. Result demonstrated that perceived stress was associated with poor sleep quality. Anxiety and depression partially mediated the association between perceived stress and poor sleep quality, explaining 73.08% of the association. This study’s structural equation model offers a useful framework for assessing mechanisms mediating associations between perceived stress and sleep quality. The findings emphases the importance of addressing psychological factors in high-pressure situations, which can exacerbate sleep disturbances among medical students. It is important to screen the level of stress, mental health problems and investigate the risk factors of sleep quality among medical students during emergency public health events.

## Introduction

When the COVID-19 outbreak that changes our daily lives. Universities were constrained to close which disturbed their regular clinical practice unexpectedly (1). COVID-19 had intensified the impact ton medical students regular teaching condition and bring a unique position in the front-line of pandemic. It brought a big challenges for medical students to adapt to the new online learning platform and an increasing worried about the delay of medical training and the risk of clinical infection during pandemic among them (2). Along with this, Medical students tend to experience higher stress levels that above for the base threshold because of the pandemic (3). However, previous study has demonstrated that higher level of stress were directly linked to poor sleep quality (4). Sleep disturbances among medical students are rapidly worsening by the impact of COVID-19 pandemic globally (5).

Stress is a high risk factor for sleep quality by affecting the sleep pattern. Previous study indicated that stress has been found interrupting sleep rhythm (low wave and rapid eye movement phases) by decreasing sleep efficiency and increasing wakefulness (6). Additionally, acute and chronic stress reactions affect the secretion of cortisol through the hypothalamic–pituitary–adrenal (HPA) axis, further affecting the changes in circadian rhythms and the quality of sleep (7). It may lead to maladaptation of the HPA axis, such as causing cortisol levels to rise and disrupting the sleep–wake cycle, further affecting the individual’s sleep quality. Among medical students, it intimated that the increasing in stress maybe due to poor sleep quality during the pandemic.

Sleep disturbance is interrelated with psychological, behavioral, physiological, and environmental factors (8). According to the cognitive model of insomnia, negative emotions (mainly depression and anxiety) can trigger people’s cognitive biases regarding stressful life events, making them excessively alert, gradually affecting sleep quality (9).However, medical students often experience long-day study, internship duration, night shifts, and high-pressure work environments, which can severely impact their sleep quality. Furthermore, Studies have reported that anxiety and depression has a clinically significant effect on sleep quality during the pandemic among adult (10).depression symptoms were shown to decrease sleep quality among medical students (11).The emotional states can negatively impact sleep quality, and sleep problems can further exacerbate mental health problems. So, it is important to screen mental health problems and investigate the risk factors of sleep quality among medical students.

Government responded to the pandemic by limiting or cancel the daily gathering which enhanced public isolation. Medical students were appear to be stressful, which not only because of the academic stress and exam pressures, but also confused about the future that they need to continue clinical practice rotations in an unknown public health event. These pressures can lead to anxiety, depression, fatigue, and other mental health problem. However, Studies were conducted that psychological impact was linked with high perceived stress among college students during the COVID-19 pandemic (12).In addition, Previous studies have reported stress symptoms related to depression, anxiety among medical students over year during the public health emergency (13).

So far, Anxiety and depression are susceptible to changes among medical students in the public health events because of the stress of academic, clinical mission, health concern and social isolation. We found that previous studies were limited in a small sample size among medical students during pandemic. More importantly, the relationship between stress, sleep and mental health were rarely among medical student in a large sample in the early stage of pandemic. The previous studies provided the worth insight on identifying the mental and health problem among medical students to provide further helpful intervention. This study aimed to clarify whether perceived stress was associated with sleep quality among medical students during pandemic. Additionally, we analyzed the perceived stress, sleep quality and mental health data through structural equation model which in order to identify pandemic-associated stress affected sleep quality through anxiety and depression among medical students.

## Methods

### Participants

This study calculated the sample size based on the Monte Carlo mediation effect statistical power analysis method. Using the standard that the statistical power of the mediation effect test was 0.95, and calculated the sample size by using M plus software to perform Monte Carlo SEM statistical power analysis (14). The final sample size obtained was 700. Taking into account the 10% recovery error, the required sample size was at least 770 people. Data were obtained from medical students enrolled in two universities in Guangdong Province, China, from January to September 2020. The inclusion criteria were (1) participating in the study voluntarily and signing the informed consent form and (2) actively attending the university. The exclusion criteria were (1) having a history of mental illness, (2) taking anti-psychotic or sedative hypnotic drugs regularly for the previous 6 months, (3) overseas students, and (4) non-attending students and those studying abroad. In total, 1,619 medical students were selected to complete questionnaires through the Questionnaire Star app, which can be used on a computer or mobile phone. Of the returned questionnaires, 1,021 were considered valid; that is, they were completed without response inconsistencies (overall effective response rate: 63.06%).

Before commencing the study, the researchers explained to the participants the purpose and significance of the investigation, that participation was anonymous, that the data obtained would be kept strictly confidential, and that the data would be used solely for research purposes. This approach helps alleviate the anonymity concerns of research participants and ensures data reliability. This study follows the principle of informed consent and anonymously saves the original information and data for the sole purpose of this study. The Ethics Committee of the First Affiliated Hospital of KY-2020-086 reviewed and approved this study (ethics review number: KY-2020-086).

### Assessment of covariates

We considered the following characteristics as potential covariates. Participants completed a sociodemographic questionnaire that included information on sex (male and female), education level (college or postgraduate or doctor), education (full-time, part-time), birthplace (rural, township, city), lunch break habit or not, accommodation or not (living arrangement or living with parents), time spent watching TV or surfing the Internet (<1 h, 1–2 h, ≥3 h), smoking or not, health status, family monthly income (≤ 2,500, 2,501–5,000, 5,001–7,500, 7,501–10,000, > 10,001), BMI (< 18.5, 18.5–23.9, > 23.9), among others. Detailed information is provided in Table 1.

**Table 1:** Demographic characteristics of medical students.

Variables	N	%
Sex		
Male	322	31.53
Female	699	68.47
Educational level		
Undergraduate	481	47.11
Postgraduate	502	49.17
Doctoral	38	3.72
Form of education		
Full-time	967	94.71
Part-time	54	5.29
Birthplace		
Rural	496	48.58
Township	258	25.27
City	267	26.15
Lunch break habit		
Yes	785	76.89
No	236	23.11
Accommodation		
Yes	816	79.92
No	205	20.08
Time spent watching TV or surfing the Internet		
< 1 h	73	7.15
1–2 h	304	29.78
≥ 3 h	644	63.07
Smoking		
Yes	22	2.15
No	999	97.85
Only child		
Yes	266	26.05
No	755	73.95
Health status		
Well	985	96.47
Chronic disease	36	3.53
Monthly income (RMB)		
≤ 2,500	218	21.35
2,501–5,000	360	35.26
5,001–7,500	204	19.98
7,501–10,000	98	9.60
> 10,001	141	13.81
BMI		
< 18.5	231	22.62
18.5–23.9	661	64.74
> 23.9	129	12.64
PSS-10	17.01	12.20–21.81
PSQI		
No sleep disturbance (PSQI <8)	727	71.20
Sleep disturbance (PSQI ≥8)	294	28.80
PHQ-9		
No depression (0–4)	490	48.00
Mild depression (5–9)	376	36.83
Moderate depression (10–14)	100	9.79
Severe depression (15–27)	55	5.38
GAD-7		
No anxiety (0–4)	516	50.53
Mild anxiety (5–9)	393	38.49
Moderate anxiety (10–14)	81	7.93
Severe anxiety (15–21)	31	3.05

PSQI, Pittsburgh Sleep Quality Index; PSS-10, Perceived Stress Scale; BMI, body mass index; PHQ-9, Patient Health Questionnaire-9; GAD-7, Generalized Anxiety Disorder-7 Scale.

### Sleep quality

The Pittsburgh Sleep Quality Index (PSQI) was used to assess sleep quality in the previous month. This index has been widely used to assess sleep quality and comprises 19 self-evaluated items and 5 other-evaluated items; of these, 18 of the self-evaluated items are used for scoring (15). These scoring items are combined into seven components: subjective sleep quality, time to fall asleep, sleep time, sleep efficiency, sleep disorders, hypnotics, and daytime dysfunction. The maximum PSQI score is 21 points, and the higher the score, the worse the sleep quality. A score > 5 points indicates sleep disturbance. In this study, Cronbach’s alpha was 0.765.

### Perceived stress

Perceived stress was assessed using the Perceived Stress Scale (PSS-10) (16), which evaluates stress levels in the last month. The Chinese version of the PSS-10 scale has high reliability and can be applied to college students (17). The scale has 10 items with total scores ranging from 0 to 40 points. The higher the total score, the greater the stress experienced by the individual. In this study, Cronbach’s alpha was 0.642.

### Anxiety symptoms

Anxiety symptoms were evaluated using the Generalized Anxiety Disorder-7 Scale (GAD-7), which is used to evaluate emotional distress in the past 2 weeks (18). The scale has seven items that are rated on a 4-point scale, yielding a maximum total score of 21 points. Scores are divided into no anxiety (0–4 points), mild anxiety (5–9 points), moderate anxiety (10–14 points), and severe anxiety (15–21 points). Cronbach’s alpha was 0.927 in this study.

### Depressive symptoms

Depressive symptoms were evaluated using the Patient Health Questionnaire-9 (PHQ-9). The PHQ-9 is a 9-item self-assessment tool based on major depressive disorders as per the *Manual of Diagnosis and Statistics of Mental Disorders (4th Edition)*. The scale has nine items that are rated on a 4-point scale (19). The maximum total score for the scale is 27, and the scores are divided into no depression (0–4 points), mild depression (5–9 points), moderate depression (10–14 points), and severe depression (15–27 points). In this study, Cronbach’s alpha was 0.905.

### Statistical analysis

To describe the data means and standard deviations were used for measurement tool data, and frequencies and percentages were used for countable data. Pearson’s correlation (normally distributed data) and Spearman’s correlation (non-normally distributed data) coefficients were used to analyze the correlation between perceived stress and sleep quality indicators. Statistical tests were 2-sided, with *p* < 0.05 indicating statistical significance.

The mediating role of anxiety and depression in the relation between perceived stress and sleep quality was analyzed with IBM SPSS Amos 24.0. We first estimated the proportion of each path coefficient and assessed the direct and indirect effects in the model. Second, we analyzed the model fit of the hypothesized equations. The goodness of fit indices considered were χ^2^/df < 5; comparative fit index, goodness-of-fit index (GFI), adjusted goodness-of-fit index (AGFI), normed fit index (NFI), incremental fit index (IFI), Tucker-Lewis index >0.90, and root mean square error of approximation (RMSEA) < 0.08, in compliance with the standards for structural equation modeling (20). By using a bootstrap test with 5,000 samples, 95% confidence intervals were calculated. A confidence interval excluding 0 indicates that the mediation effect is significant.

## Results

### Demographic characteristics

The demographic information of participants and the scores of perceived stress and sleep quality are shown in Table 1. In total, 1,021 medical students were enrolled in this study; 322 (31.53%) were male, 699 (68.47%) were female, 481 (47.11%) were undergraduate students, 502 (49.17%) were postgraduate students, and 38 (3.72%) were doctoral students.

### Correlation analysis

The correlations between the four variables are presented in Table 2. Perceived stress was positively related to sleep quality (*r* = 0.305, *p* < 0.01), anxiety (*r* = 0.426, *p* < 0.01), and depression (*r* = 0.401, *p* < 0.01).

**Table 2:** Correlations between sleep quality, perceived stress, anxiety, and depression.

	Sleep quality	Perceived stress	Anxiety	Depression
Sleep quality	1	–	–	–
Perceived stress	0.305**	1	–	–
Anxiety	0.503**	0.426**	1	–
Depression	0.576**	0.401**	0.811**	1

**p < 0.01, *p < 0.05.

### Measurement model

**Figure 1:**
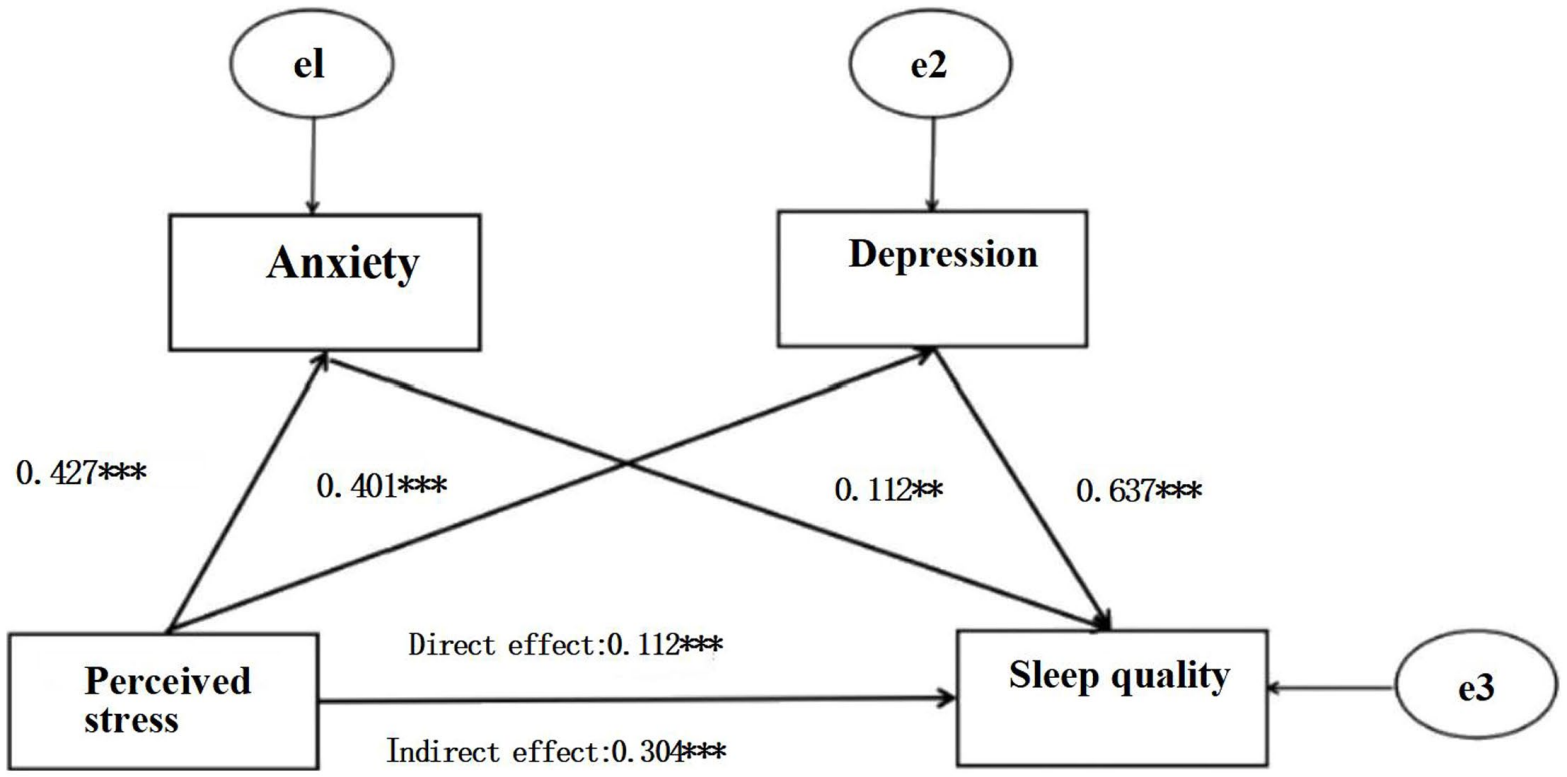
Serial mediation model for perceived stress, anxiety, depression, and sleep quality. Path coefficients are shown in standardized regression coefficient form. **p < 0.01, ***p < 0.001.

Sleep quality was entered as the dependent variable, anxiety and depression as the mediating variables, and perceived stress as the independent variable. As shown in Figure 1, perceived stress had a direct positive effect on sleep quality (*β* = 0.112, *p* < 0.001). The model showed appropriate fit, as per the following fit index results: χ^2^/*df* = 2.985, *p* < 0.005, AGFI = 0.969, GFI = 0.985, RMSEA = 0.043, NFI = 0.976, IFI = 0.984.

### Structural model

The path coefficients of the three indirect and direct paths were significant, as shown in Tables 3, 4. The results of the path analysis showed that perceived stress had a positive effect on anxiety, as indicated by the path coefficient (*β* = 0.427, *p* < 0.001). Perceived stress had a positive effect on depression (*β* = 0.401, *p* < 0.001), depression had a positive effect on sleep quality (*β* = 0.637, *p* < 0.001), and anxiety had a positive effect on sleep quality (*β* = 0.112, *p* = 0.018). The first indirect path was that the effect of perceived stress on sleep quality was mediated by anxiety, with an effect size of 0.048. The second indirect path was that the effect of perceived stress on sleep quality was mediated by depression, with an effect size of 0.043. The third indirect path was that the effect of perceived stress on sleep quality was significantly mediated by both anxiety and depression, with an effect size of 0.213. Furthermore, a direct effect of perceived stress on sleep quality was demonstrated, with an effect size of 0.112.

**Table 3:** Standardized path coefficient analysis results of the model.

Variables			B	Non-standardized coefficient	Standardized coefficient	S.E.	*p*
Perceived stress	→	Anxiety	0.427	0.380	0.427	0.025	*p* < 0.001
Perceived stress	→	Depression	0.401	0.069	0.067	0.021	*p* < 0.001
Perceived stress	→	Sleep quality	0.112	0.014	0.144	0.003	*p* < 0.001
Depression	→	Sleep quality	0.637	0.061	0.637	0.005	*p* < 0.001
Anxiety	→	Sleep quality	0.112	0.012	0.112	0.005	0.018

S.E, standard error.

**Table 4:** Results for the mediating effects of anxiety and depression.

Variables	B	S.E.	Bias-corrected 95% CI			95% CI			Effect size
			Lower	Upper	*p*	Lower	Upper	*p*	
stdind1	0.048	0.022	0.007	0.094	0.018	0.006	0.093	0.021	11.54%
stdind2	0.043	0.014	0.018	0.071	0.001	0.017	0.069	0.001	10.34%
stdind3	0.213	0.022	0.173	0.259	0.000	0.171	0.258	0.000	51.20%
Total indirect effect	0.304	0.025	0.255	0.351	0.000	0.255	0.351	0.000	73.08%
Direct effect	0.112	0.050	0.016	0.215	0.019	0.014	0.213	0.021	26.92%
Total effect	0.416	0.062	0.299	0.542	0.000	0.296	0.538	0.000	100.00%

stdind1, perceived stress–anxiety–sleep quality; stdind2, perceived stress–depression–sleep quality; stdind3, perceived stress–anxiety–depression–sleep quality; S.E, standard error; CI, confidence interval.

## Discussion

This study investigated the relationship between perceived stress, sleep quality, anxiety, and depression among medical students in larger sample during the COVID-19. We found that anxiety and depression mediated the relationship between perceived stress and sleep quality. Additionally, the present study demonstrated that perceived stress directly affected sleep quality that brought sleep difficulties among medical students that was similar as hypotheses and prior reaches of pandemic (21). On the other hand, Perceived stress also indirectly affected sleep quality by the mediating effect of anxiety and depression. These findings are comparable to those of previous studies on medical students, which found that mental distress (including stress, anxiety, and depression) directly predicts poor sleep habits (22).

Our study profound medical students were in a stressful condition, not only for academic pressure, but also adapt to the new study surrounding, unique lifestyle during the pandemic period. Prior research indicated that medical students adopted a pattern of ‘sleeping late and getting up early’ to meet academic demands and that this lifestyle affected their sleep quality, resulting in daytime sleepiness and insomnia (23). In particular, At the beginning of pandemic, it had been reported that more under-graduated students feel more stress because of the doubts about the future (24). Besides, One study in America found that the increasing stress disturbed college students’ sleep duration and led to poor sleep quality (25). Moreover, the change of study style including online environment, coursework delivery and social contact that is difficult for students to adjust themself at the first time which established consequences on stressful condition and poor sleep. In addition, it has reported similar results among adolescents under long-term stress; affected individuals’ sleep quality and responsiveness, resulting in more sleep problems (26). Hence, It is important to identified the severity of sleep quality and perceived stress during public health events to ensure retain a healthy lifestyle and alleviate the stressful condition among them.

Base on structural model, our study has identified that anxiety and depression mediated the relationship between perceived stress and sleep quality. Therefore, stress may not be driven to poor sleep quality by directly, and emotional reactions may play an important mediating role in contributing toward sleep disturbance among medical students during pandemic. The links between perceived stress, poor sleep quality, depression and anxiety were well verified. From a neurophysiological perspective, stress responses can cause emotional changes, and emotional stimulation affects sleep through the interaction between brain regions that process emotions and those that control sleep and arousal, disrupting the circadian rhythm balance (27). Research evidence suggested that the indirect path from stress to sleep quality through psychological factors.

Several potential mechanisms may explanation the association between perceived stress, poor sleep quality, depression and anxiety. First, Previous study reported that individual experienced studies-related stress events will increase anxiety symptoms through the activation of HPA axis and led to sleep disturbance (28). Second, according to Lazarus’ stress and coping theory, individuals who have been in high-pressure situations for a long time cannot adapt to or deal with working under pressure, which causes them anxiety and affects their sleep quality (29). Furthermore, A systematic review found that emotion regulation is closely related to sleep duration among adolescents (30). Hence, it may be that, through perceived stress, depression can negatively affect the rapid eye movement phase, disrupting the sleep rhythm and affecting the normal sleep cycle, finally affecting an individual’s sleep quality.

The results of the mediating effect test indicated that medical students’ stress to the impact of sleep quality was mediated by mental health during COVID-19 pandemic. Recent data conducted in Spain demonstrated that university students had a high level of stress linked with depression and anxiety when city lockdown (31). Students showed strongly concern about the health, academic and social situation. The result is consistent with our study. Another study concluded that healthcare workers have a high related between perceived stress, depression and anxiety (32). Indeed, stressful life events was pointed out as risk factors for depression. In addition, a finding supported that someone who were depression, they were sensitive to sudden events, and contribute to stress during COVID-19 (33). With this background, it is important to concerned about public heath events maybe the sensitive incident bring a lot stress and due to the depression and anxiety symptoms among medical students.

Worsened sleep may result in adverse effects. That is, sleep disturbance is closely related to emotional stability. Previous reviews reported that psychological distress was strongly associated with sleep disturbances among healthcare professionals, the general population, and COVID-19 patients during pandemic (34). Research has found that the relationship between emotional stability and insomnia symptoms is mediated by anxiety and depression (35). Negative emotions may lead to unhealthy behaviors, such as excessive worrying about the future, excessive caffeine intake, and daytime sleepiness, among others, due to sleep dysfunction. In addition, this study supported that higher anxiety levels were associated with poorer sleep quality, which is consistent with previous studies during COVID-19 among Chinese participants (36). In particular, because of the remote learning, students lacked of social connection, outdoor activities, more time in using internet tools, the uncertainty of infection and COVID-related worries that interfere the sleep quality.

### Limitations

The current study has several limitations. First, causal relationships could not be verified because of the study’s cross-sectional design and SEM are recommended for prospective studies. Besides, longitudinal designs are needed to verify those mental symptoms, stress condition and sleep quality. Second, using convenience sampling is non-optimal, as it may lead to a non-representative study population, and causality cannot be confirmed. Furthermore, only two universities were selected as the study sites; hence, future researchers should use larger, more representative samples. Moreover, only a self-assessment questionnaire was employed to measure sleep quality in this study. Other objective measurements should be used to investigate sleep quality in future research.

## Conclusion

The findings of the present study suggest that associations between perceived stress and sleep quality can be explained by anxiety and depression symptoms. The findings contribute to an understanding of the psychological factors (anxiety and depression symptoms) linking perceived stress and sleep quality among students during COVID-19. We suggest that further research is needed to comprehensively evaluate stressful situations, sleep quality, and psychological factors among students when there is outbreak public health events. We also acknowledge that medical students should screen and take measures to cope with pressure and focus on relieving anxiety and depression symptoms to improve sleep quality during the unexpected emergency public health disease.

## Data availability statement

The raw data supporting the conclusions of this article will be made available by the authors, without undue reservation.

## Ethics statement

The studies involving humans were approved by This study was approved by the hospital’s Ethics Committee (Reference number: KY-2020-086). The studies were conducted in accordance with the local legislation and institutional requirements. The participants provided their written informed consent to participate in this study.

## Author contributions

WH: Writing – original draft, Writing – review & editing. XW: Data curation, Writing – review & editing. YL: Data curation, Investigation, Writing – review & editing. CL: Formal analysis, Funding acquisition, Project administration, Writing – review & editing.
